# Composition of Plastic Fractions in Waste Streams: Toward More Efficient Recycling and Utilization

**DOI:** 10.3390/polym11010069

**Published:** 2019-01-05

**Authors:** Ville Lahtela, Marko Hyvärinen, Timo Kärki

**Affiliations:** 1 LUT RE-SOURCE Research Platform, LUT University, P.O. Box 20, FI-53851 Lappeenranta, Finland; 2 Fiber Composite Laboratory, LUT University, P.O. Box 20, FI-53851 Lappeenranta, Finland; marko.hyvarinen@lut.fi (M.H.); timo.karki@lut.fi (T.K.)

**Keywords:** circular economy, near-infrared (NIR), plastic, separation, waste

## Abstract

Reuse of materials is a significant global goal that contributes to sustainable development. Polymer-specific plastic identification from the waste stream is examined in this study to achieve environmentally optimistic reuse of plastic material in secondary applications. Two diverse waste streams, 86.11 kg of construction and demolition waste (CDW) plastic and 57.74 kg of mechanically sorted plastic, were analyzed by using a handheld tool whose identification technology was based on the near-infrared spectrum. The study indicates a significant effect of human and single fraction on manual separation. The polymer composition in the plastic waste stream varied depending on the source, but the most common plastic grades, polypropylene (PP) and polyethylene (PE), were represented in every waste stream. The waste stream also included unidentified and unfavorable wastes, which indicates that identification of the plastic fractions is needed and more studies should be done in this field in the future.

## 1. Introduction

Having zero-waste nations is a global goal, and steps toward the reuse and recycling of material should be taken to contribute to this aim. The majority of life-cycle assessment (LCA) studies have concluded that recycling polymer plastic waste fractions is generally the environmentally preferred treatment option compared to incineration and landfilling [1]. In addition, the reuse of plastic reinforces the viewpoint of a circular economy, which has been a universal trend in recent years.

The favorable features of plastic contribute to its utilization in a wide range of applications, such as packaging, and in automotive and electronics segments. The building and construction segment is one of the biggest plastic users in Europe, as this segment uses 19.7% of European plastics [2]. The great demand of plastic also affects the amount of generated plastic waste. The main sources of post-consumer waste plastics are municipal solid waste (MSW), construction and demolition waste (CDW), waste from electric and electronic equipment (WEEE), and end-of-life vehicles (ELV) [3]. For example, a modern car consists of 12–15% plastics, and the amount of plastics has increased in the car industry due to the demands of lightweight and fuel-efficient vehicles [4].

Waste management is influenced strongly by the waste hierarchy, which recommends a priority order to reduce environmental impacts: prevention, reuse, recycling, recovery, and disposal [5]. Industrialized economies have existing legislative policies that include 3R (reduce, reuse, recycle) initiatives in CDW management. Effective examples can be found in Europe, where reuse and recycling of CDW have been developed effectively since the 1990s, due to taxation, charging fees, ban on landfilling, and laws requiring CDW separation [6]. Studies related to the field of CDW management have received increasing attention around the world in the 2000s [7]. There have also been some challenges in achieving sustainable CDW management. One challenge for sustainable management with regard to CDW has been weak or ineffective legislation. Transitory and developing economies do not have explicit laws concerning generation, management, and disposal of CDW, and this hampers the categorization of CDW. For example, the inadequacy of CDW management has been clearly detected in the case of earthquakes. In addition, the reframing of CDW as a source of raw materials instead of solid waste has been a challenge. Even though CDW material can be a rich urban mine, it may also be time-consuming and economically unsustainable [6]. Good geographic location is a strength in construction waste management [8]. For example, Paranhos et al. [9] have found that 50 km is the maximal distance for transporting CDW. Therefore, waste plastic should be utilized locally instead of exportation. However, a huge amount of waste plastic is being exported due to unstable domestic markets. For example, about 50% of the Finnish annually recycled waste plastic is estimated to be exported due to a low domestic demand [10].

The composition of CDW is somewhat unclear, and studies related to it are few in number, even though a minimum of 70% by weight of CDW must re-used or recycled in the area of the European Union by 2020 [11]. The plastic material contains a wide range of polymer types, and its composition within CDW is also unclear. The polymer types are identified by a seven-phase coding system, which allows efficient separation for recycling [3]. A recent study showed that the composition of plastic bags plays a major role in environmental and health problems, such as chlorine (Cl) content in polyvinyl chloride (PVC) plastic [12]. PVC is a widely utilized material in the construction and demolition (C&D) sector, so its share in the waste stream should be investigated due to its essential role in recycling processes [13]. One piece of PVC in the midst of a thousand other polymer pieces can ruin the entire batch [14]. The construction industry produces about 35% of landfill waste as a global average [15], and almost 30% of plastic waste will end up in landfill, which is still an option in several European countries [2]. However, the removal of plastics from landfill should make significant economic and environmental sense. The European Commission adopted a strategy on plastics at the beginning of 2018, which will create new opportunities for innovations in the field of plastic waste [16]. 

The aim of this study was to assess the composition of plastic waste. Current concerns relating to the environment being filled with plastic create need for new recirculation ideas for plastic waste. Research into plastic waste is important because the structure of plastics should be analyzed before innovative utilization. This information removes barriers to new applications for secondary plastics. Plastic waste was collected from different sources, which consisted of manually separated CDW and mechanically separated plastic wastes from sorting plants. The composition of plastic was identified with near-infrared (NIR) analyzer equipment.

## 2. Materials and Methods

Two different material streams from varied stakeholders were used in the experiments of plastic identification. Stream 1 was obtained from a waste company, from which six loads of construction and demolition waste materials were received. The plastic materials were separated manually into categories based on the source separation model. Stream 2 was obtained from waste companies where plastic waste materials were separated mechanically by optical sensors. The waste companies behind the streams were jointly owned by municipalities which had decades of experience in the waste management sector. The studied amount of materials were 86.11 kg for manual sorting and 57.74 kg for mechanical sorting. The mechanically sorted stream also included a sample batch (1.10 kg) for smaller-sized particles to confirm the reliability of the results.

The identification of plastic waste materials was analyzed with a handheld NIR tool (Thermo Scientific microPHAZIR PC, Thermo Fisher Scientific, Waltham, MA, USA) that identifies material in less than three seconds in the spectral range of 1600–2400 nm without special sample preparation. The share per component in the material stream was determined based on the weight. 

## 3. Results

### 3.1. Materials

The received amount of manually separated CDW from Stream 1 included 18% of plastics consisting of over 150 kg of classified plastic material, but it also included weighty and dark rubber mats that were removed from the test materials because they could not be categorized as plastics with certainty. Therefore, the total amount of plastic waste in the manually separated CDW for the test was 86.11 kg. 

Stream 2 included mechanically separated plastic waste from a sorting plant. The obtained amount of this stream was approximately one cubic meter, including particles of various sizes. In this study, bigger particles with widths being at least approximately 50 mm were identified, and the studied amount of material was 57.74 kg. In addition, a smaller-sized sample batch of 1.10 kg was analyzed to ensure that the identification results were actual and could be confirmed regardless of particle size.

The share of plastic within CDW is unknown, and the values have varied greatly at the global level from 1 to almost 10% in previous studies [17,18]. The more commonly studied MSW globally includes 8–12% plastic waste [19]. The rough initial sorting in the case of manually separated CDW (share of plastic from 150 kg to 86.11 kg) demonstrated that manual separation clearly depends on human influence and motivation.

### 3.2. Composition

The composition of each plastic material in the various waste streams is presented in Table 1. A sample batch (2*) from a mechanically sorted stream is marked individually in Table 1. It can be seen that the polypropylene (PP) fraction represents the largest volume of a certain polymer in plastic waste, especially in the case of mechanically separated plastic waste materials.

**Figure 1:**
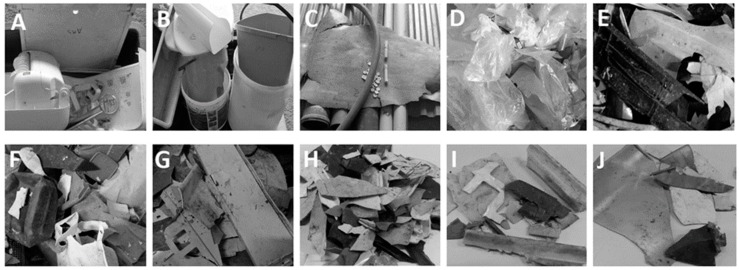
Illustrative examples of ABS (**A**), PP (**B**), PVC (**C**), and PE (**D**) in manually sorted CDW. PP (**E**), PE (**F**), and PS (**G**) after mechanical sorting, together with smaller-sized PP (**H**), PE (**I**), and ABS (**J**).

The sample batch from mechanically sorted plastic represented the whole composition of the plastic group quite well, and the smaller particles were mainly congruent with the main group. The clearest exceptions were visible in the case of PE and ABS polymers, but the exceptions were not dramatically different. Figure 1D,F shows that the PE polymer is a commonly used material in plastic films and consumer products, such as containers. Perhaps due to the flexible feature of PE, the consumer products remained intact during the process steps, which can explain their bigger share in the mechanically sorted plastic batch.

## 4. Discussion

The manually separated plastic waste contained a wider spectrum of plastic fractions. The largest polymer was ABS, which can be explained by one sample (trailer canopy) in the waste stream. Without the canopy, the amount of ABS would be approximately 25 kg less. In this situation, the share of ABS could be approximately below ten percent, and the biggest plastic fraction would be PP, similar to the mechanically separated streams. This also corresponds to the situation in European plastic markets, where PP is the most common polymer. However, in the C&D segment, PVC is a certain predominant polymer, as it is used for things like window profiles and floor coverings [2,3]. This may be quite problematic in the future because it is thought that PVC is a potential source of toxic chemical release in pollution emissions [12]. Of course, regional differences have a remarkable influence. For example, the total share of PVC production has increased by 23%-units in Asia in two decades, while concurrently, the corresponding shares of North America and Western Europe have decreased by 9 and 12 percent units, respectively. In turn, PVC consumption increased by 14.8% in Central Europe in the period 2002–2007 [20]. From the viewpoint of window frames, PVC is a cost-effective material, and over 40% of window frames are made from PVC plastic in Europe. However, regional differences may again be significant, such as wood-aluminum representing 80% of total window-frame production in Finland. The product life of PVC windows are approximately a decade lower compared to wood-metallic window-frames [21,22]. Overall, according to Ciacci et al. [23], PVC is a young material and its in-use stock is likely to increase remarkably. The ABS polymer also demonstrates how significant the effect of an individual sample can be in the results. Another exception was a minor amount of PE fraction in the manually separated CDW compared to the mechanically separated streams. The minor share of PE can be explained in the form of PE polymers because it is often used in plastic films (Figure 1D), and its effects on the weight is small even though the volume can be high. Additionally, it must be taken into account that film plastics substantially decrease the separation efficiency [24]. Polyamide (PA) clearly appears as a separate fraction within CDW, while it has not been noticeable in other streams. The remarkable share of PA in CDW might be explained by its good mechanical properties [25].

ABS is quite often used for things like computer keyboards and toys [26], and it may become shredded to small parts during processing. However, recycled ABS in post-use contributes to unfavorable properties compared to virgin materials [27].

The used sensor did not identify 15% of the materials, except for the test group. The NIR technology is unable to identify dark materials, and the shares of them in the manually separated CDW and industrially separated plastic were 30.39% and 49.27%, respectively. From the recycling point of view, dark color should be avoided in plastic materials because its identification is currently quite challenging. Overall, the identification of polymers within the waste stream is still challenging, and needs to be studied more in the future. In addition, the design of products and identification of technology for waste objects must be taken into account.

Tam and Tam [28] state that the recycled plastic from CDW can be utilized in new versatile products. Overall, PP and PE are often identified as polymers that contribute to the set targets of the re-use and recycle of CDW because PP and PE release less emissions during recycling compared to ABS and PVC polymers, for example. In addition, recycled PP can be easily improved by compounding with additives [29]. However, the usability of recycled plastic depends on its purity and recycling technology, including several steps [30]. Therefore, future studies should be focused on the methods that simultaneously cleans the material together with separation. The utilization of recycled plastic is in agreement with European governmental regulations, where the target is to achieve 70% of the recycling or reuse rate. A certain problem in this process might be the availability of CDW, because there are not enough reception facilities for CDW.

## 5. Conclusions

In this study, the characterization of plastic waste was assessed to find out whether it could be used as a potential raw material in reuse applications. Analysis of plastic is a novel type of work that has not been studied extensively as of date. It was found that a single waste fraction could affect the result of the composition remarkably, meaning that an inhomogeneous material stream can be troublesome from the viewpoint of sorting and recycling. PE and PP polymers are the most common plastic grades in the waste stream, which will offer an opportunity to reutilize the material, strengthening the idea of circular economy at the same time. The C&D waste stream contains a wide spectrum of materials, also including harmful grades from the point of view of further processing. In the worst-case scenario, a harmful polymer is manufactured as a dark product, which the technology currently available is unable to separate. This kind of a scenario should be noticed before manufacturing, taking account of the eco-design aspect. 

## Figures and Tables

**Table 1:** Plastic polymers, acrylonitrile-butadiene-styrene (ABS), polyamide (PA), polycarbonate (PC), polyethylene (PE), polyethylene terephthalate (PET), polymethyl methacrylate (PMMA), polypropylene (PP), polystyrene (PS), and polyvinyl chloride (PVC) shares (%) in the waste stream. Stream 1 = manually sorted construction and demolition waste (CDW); Stream 2 = mechanically sorted CDW; Stream 2* = mechanically sorted sample.

Stream	ABS	PA	PC	PE	PET	PMMA	PP	PS	PVC	Un ^1^/d ^2,^*
1	33.91	9.44	0.52	8.44	1.10	0.64	21.45	0.09	9.69	14.71/30.39
2	0.74	-	-	28.42	0.12	-	48.27	5.66	0.05	16.73/49.27
2*	4.91	-	-	6.55	0.32	0.61	53.01	3.73	0.15	30.72/62.00

^1^ Unidentified, ^2^ Dark-colored polymers, * share of dark color material in unidentified material.
